# The structure of spontaneous speech changes in Alzheimer’s disease: Crosslingual evidence from English and Greek

**DOI:** 10.1371/journal.pone.0324270

**Published:** 2025-05-22

**Authors:** Hong Jiang, Zhengwei Chen, Yu Liu, Chun Yang, Xiaofeng Yuan, Rui He

**Affiliations:** 1 Zhuhai People's Hospital (The Affiliated Hospital of Beijing Institute of Technology, Zhuhai Clinical Medical College of Jinan University), Zhuhai, China; 2 Faculty of Medicine, Macau University of Science and Technology, Macau, China; 3 Jinlin Medical College, Jilin, China; 4 Guangzhou Railway Polytechnic, Guangzhou, China; 5 Grammar and Cognition Lab, Department of Translation & Language Sciences, Universitat Pompeu Fabra, Barcelona, Spain; University of Missouri Columbia, UNITED STATES OF AMERICA

## Abstract

Impairment in the semantic domain is prominent in Alzheimer’s Disease (AD). We analyzed spontaneous speech in English from 148 people with probable AD (pAD) and 143 controls, and aimed to replicate these findings in a smaller Greek dataset of 28 controls and 26 pAD patients, using different language models comparatively. Static models (fastText) represented non-contextual meaning via encoding words as static vectors, while contextual models (BERT) represented the contextual meanings sensitive to syntactic structure. These models calculated semantic similarity at two levels: local similarity (between adjacent words/tokens) and global similarity (across all word/token pairs). Generative contextual models (Mistral) additionally quantified token probability within context, thereby indicating the unexpectedness in speech progression. Given that contextual meaning is syntactically sensitive, we introduced averaged dependency distance as an indicator for formal syntactic complexity. Moreover, bimodal models were introduced to evaluate how speech reflected picture-based stimuli. Results showed significant increases in global semantic similarity in the pAD group, as measured by both fastText and BERT models, which co-occurred with enlarged picture-speech semantic distance and increased in speech perplexity. Only the fastText-based global semantic similarity, which captured the contraction in conceptual semantic space, correlated with the overall cognitive decline in the AD populations. These findings together indicates that semantic space changes in AD differed across different forms of meanings and thus points to the necessity of distinguishing these forms to raveling the underlying mechanism.

## Introduction

Spontaneous speech has been shown to be an informative digital biomarker for understanding neurocognitive degeneration in the Alzheimer’s disease (AD). Alternations in speech profiles can occur throughout the spectrum of cognitive decline including before clinical disease onset [1,2]. Evidence has revealed atypical speech profiles at the very early stage of AD [3,4], in mild cognitive impairment (MCI) [5–10], in healthy older adults with subjective cognitive decline (SCD) [11,12], and even in cognitively unimpaired older adults with positivity in biomarkers [13,14]. Across different domains in speech and language, impairments in the semantic domain are found to be among the most prominent [15,16] and to be powerful indicators for detecting cognitive decline in the AD spectrum [11].

The construction of meaning in discourse integrates two distinct types of meaning. On one end stands the lexical conceptual meaning representing our semantic knowledge of the world, e.g., *king, queen, apple,* or *run*. These lexical concepts exist in the semantic space, ready to be retrieved for constructing a discourse where these concepts are utilized to generate references to entities and events in the world. For example, *John caught a cold during his study in London* captures a specific event about a named person at a certain time and place. Meaning at this other end is referential and grammatical, and encapsulates thoughts to be shared in communication [17]. Integrating the lexical meaning and referential meaning involves the propagation from lower-order cognitive representations (such as visual, orthographic, and phonological properties) to higher-order representations (such as contextual meaning and speech acts) [18], which aligns with the large-scale hierarchical organization of the human cortex [19,20] and serves as a potential indicator for impairment in the cognitive profile [21,22]. Traditional psychological experiments have provided indirect evidence for impairments in both types of meanings (e.g., naming errors in AD have been found to stem from both semantic knowledge deficits and reference matching issues [23]). Recent advances in natural language processing (NLP) techniques have empowered a more naturalistic and automatic exploration of changes in this dimension of language in AD through the lens of spontaneous speech [11,15,24–27].

Among the computational measures, semantic distance serves as a crucial descriptor of the semantic spaces aforementioned. In a conceptual semantic space, lexical concepts are interconnected through statistically measurable associations such as co-occurrences. The co-occurrence patterns of a word serve as a latent value to explain its meaning [28]. Such a distributional framework of meaning forms the foundation of static language models such as word2vec [29]. In these models, each word is represented as a high-dimensional vector storing distributional information from large corpora, with the angle between two vectors determining their similarity. Analyzing the semantic similarity between consecutive word pairs (i.e., local similarity) or all word pairs (i.e., global similarity) can provide valuable descriptions on the structure of the semantic space. Paula et al. carried out semantic similarity analysis on verbal fluency data, where participants were instructed to generate lists of lexical concepts within a specific category, and found increases in the similarity among produced concepts [30].

Despite its power in modeling meaning, the distributional hypothesis is naturally weak in explaining the other end of meaning – reference. In a distributional model, the meaning of a word is simply represented by the co-occurrence patterns, leaving out the relation between the linguistic words and the external world [28]. The referential meaning is produced necessarily when grammar comes into play, connecting words hierarchically and linearizing into a sequence so as to form sentential and discourse meaning, and when there are external entities and/or events in the outside world to refer to. For example, we only know the truth value of *The boy owns a piano.* necessarily when the lexical concepts *boy, own,* and *piano* are organized in this grammatical structure with the determiners and the tense marker, and when we know which boy it refers to. The first missing link of including hierarchical syntactic relationship can be approximated with contextual language models, where the embedding of a word is not only decided by the co-occurrence patterns learned from the training corpora, but with regard to the actual context it situates as well. BERT [31], one of the most prominent large language models for natural language understanding, has been noted to progressively acquire the linguistic information in its internal transformer architecture, from the first layers sensitive to superficial features such as sentence length and word count, via the middle layers more sensitive to the formal syntactic structures, and towards the final layers more sensitive to semantic information [32]. There is little evidence that static language models trained with limited contextual information can also capture the hierarchical syntactic structure of a sentence. In He et al. [21]’s work, only local similarity measures derived from contextual models, not static models, related to formal syntactic complexity, and the contextual local similarity measures change in a different direction in populations on the psychosis spectrum as compared to the static similarity measures. These results empirically validate the idea that the conceptual and referential meanings should be distinguished into two kinds. They also support the application of contextual models to represent word meaning with high sensitivity to syntactic hierarchy within meaningful grammatical units, such as sentences, which moves significantly towards the referential end of meaning.

Though the inclusion of syntactic information empowers contextual models to capture referential meaning to a certain degree, there have been long-lasting arguments for syntax preservation in AD pathology [33–36], questioning the necessity of making such distinguishment in the AD context. However, recent literature suggested that such a preservation only took place at a superficial level where language is viewed as a linear sequence of words [37], while language processing is beyond linear word production [38] but follows a hierarchical structure of formal syntax [39]. Finer-grained investigation of the hierarchical syntactic properties revealed the existence of syntactic decline in AD pathology [3,37]. Thus, there is reason to assume that the semantic structures of conceptual and referential spaces could change differently in AD pathology.

Contextual models, unlike the static models, are not specifically designed to capture semantic relations exemplified by analogies like *King* - *Man* + *Woman* = *Queen*. Instead, they predict the probability of a token in its position based on the vectorized representations of the context. The range of the contexts includes both the preceding and following contexts for masked models, while generative models consider the previous context only, as they are designed to mimic the superficial linear process of speech production, where words are produced sequentially. Although it is widely accepted that language production transcends such a linear procedure and follows a more intrinsically hierarchical structure, these generative models, represented by GPT and Mixtral, stand out among the most powerful linguistic tools in the current world. Predicting the probability of a token in its contexts offers a different approach from semantic similarity to approximating semantic structure at the referential level, introducing an information-theoretic perspective on language production. By considering the entire discourse as a system and the comprising words as internal events, the probability of each event can then be measured by contrasting each observation from actual speech with predictions made by language models, indexing the semantic unexpectedness of each word. The overall unexpectedness of the discourse is then computed by averaging the log-likelihood of every word, known as cross entropy, while the 2-based exponentiated value of the cross entropy is termed perplexity (PPL). PPL serves as a more intuitive measure than entropy itself, equating to the number of possible outcomes in a random system. For instance, while the entropy of casting a fair six-sided die is approximately 2.585 bits, the PPL equates to six. PPL has proven to be a valuable metric for investigating the loss of contextual semantic predictability in psychosis [21], and for classifying AD patients from healthy controls [40]. In the latter work, Colla et al. retrieved PPL of spontaneous speech using the English GPT2 model for classification purposes. However, they did not report the direction and statistical significance of changes in the AD pathology. Given that GPT2 is a relatively small model released for experimental purposes, applying a larger and more advanced model could further validate these findings and address the gap in statistical descriptions of PPL changes in the AD pathology.

Moreover, examining relationships within language alone is insufficient for exploring the structure of meaning in speech, as language exists and interacts with the real world. Connecting language to its real-world references is essential for gaining deeper insights into referential meaning. Spontaneous speech in AD studies is mostly elicited by describing pictures (i.e., visual stimuli), making these pictures the natural “anchor” for speech in the real world. The similarity between speech and the picture in AD was majorly explored by the number of picture-related elements. For the Cookie Theft picture, studies have identified 23 information units (e.g., cookie, jar, boy, girl, and mother) [41]. It has been observed that less information units were mentioned in the speech from AD patients [42]. However, this method is not automatic and cannot be generalized to other images, not even to a new version of the Cookie Theft picture that includes colors and an additional figure of the mother who is tending the garden. Therefore, incorporating a visual-language model would be beneficial in understanding the referential meaning structure in language. CLIP (Contrastive Language-Image Pretraining) is one such model that hypothesizes the visual and textual space majorly share similar structure and learns the mapping between these two modalities from the training data [43]. Image-text alignment has been found effective in grouping patients with probable Alzheimer’s Disease (pAD) from healthy older adults [44], indicating potential changes in the distance between the visual and textual modalities.

Efforts have now been made to quantify semantic similarity and relevant changes in AD pathology using spontaneous speech. Using static language models, Burke et al. found that patients with pAD showed similar semantic similarity among consecutive word pairs but the lower similarity between each word and the averaged embedding across all words as a proxy for the topic [45]. We aimed to expand the local similarity analysis to investigate the changes in structure of meaning in pAD. Our metrics include: (1) local semantic similarity from static language models excluding words with high frequency but little conceptual meaning, such as pronouns and determiners in the analysis of conceptual semantic space; (2) local semantic similarity from contextual models to explore changes at the referential level; (3) quantify the perplexity of produced speech;(4) analyze the similarity between the visual prompts and verb descriptions using a bimodal model, instead of creating an artificial topic by averaging the word embeddings, as the picture itself functions as the external anchor of the speech; and (5) examine the formal aspect of structural changes, i.e., the syntactic hierarchy. We first analyzed the changes in semantic space in the pAD population in an English dataset comprising 143 controls and 148 patients. Then, to validate the cross-lingual generalizability of semantic structural changes, we replicated these analyses in a Greek dataset with 28 controls and 26 patients. We predicted alternations in semantic structure could be observed in pAD patients and could be found in both datasets.

## Methods

### Datasets

We used the ADReSS-M speech data available from Dementia Bank, which contains speech recordings from normal controls (NC) and pAD patients in English and Greek (https://dementia.talkbank.org/ADReSS-M/) [46]. The use and analysis of data for the present study was in adherence to all ground rules including the ethical issues applied to Dementia Bank (https://talkbank.org/share/). Participants issued informed consent form which was approved by the local Institutional Review Board of data contributors. All data were fully anonymized before we accessed the data at 20 April, 2024. Age, sex, education, and the Mini-Mental State Examination (MMSE) scores of the subjects are reported in Table 1. Missing values for education in the English dataset were filled with the averaged education in NC, as they were all NCs. One NC didn’t have an MMSE score in the Greek dataset, which was filled with the averaged MMSE score of the Greek NC group. Inclusion and exclusion criteria can be referenced in the challenge description paper [46]. Speech samples in both the English and Greek datasets were obtained through a picture description task, though the visual stimuli are not identical. The English subjects described the Cookie Theft picture while the Greek subjects described a picture representing a lion lying with a cub in the dessert while eating. Details on the data collection, speech elicitation, and experiment design can be found in the challenge description paper [46]. We employed whisper (version: large-v3) for transcribing the speech recordings into text for the subsequent semantic analysis [47]. All relevant scripts are available on https://github.com/RuiHe1999/sem_space_AD.

**Table 1:** Descriptive variables of the English and Greek datasets.

Dataset	Variables	NC	pAD	Test	*p*	Effect size
English	Number	143	148	/	/	/
	Age	66.061 ± 6.310	69.377 ± 6.884	Mann-Whitney	<0.001	-0.283
	Sex	65.217%	64.754%	χ^2^ test	0.940	0.005
	Education	13.983 ± 2.377	11.967 ± 2.635	Mann-Whitney	<0.001	0.428
	MMSE	28.965 ± 1.169	17.844 ± 5.482	Mann-Whitney	<0.001	0.976
	Word Number	57.678 ± 27.675	51.525 ± 28.096	Mann-Whitney	0.013	0.187
	Subword Number	173.322 ± 77.964	178.221 ± 95.734	Mann-Whitney	0.816	0.018
	Word TTR	0.804 ± 0.092	0.753 ± 0.122	Mann-Whitney	<0.001	0.258
	Subword TTR	0.499 ± 0.084	0.447 ± 0.098	Mann-Whitney	<0.001	0.318
Greek	Number	28	26	/	/	/
	Age	66.571 ± 7.275	72.737 ± 6.826	Mann-Whitney	0.004	-0.459
	Sex	71.429%	73.077%	χ^2^ test	0.893	0.018
	Education	12.000 ± 4.028	9.500 ± 3.922	Mann-Whitney	0.034	0.334
	MMSE	28.821 ± 1.156	20.962 ± 4.574	Mann-Whitney	<0.001	0.975
	Word Number	29.429 ± 15.464	30.192 ± 14.702	Mann-Whitney	0.795	-0.043
	Subword Number	63.393 ± 33.065	62.577 ± 31.323	Mann-Whitney	1.000	0.000
	Word TTR	0.875 ± 0.152	0.834 ± 0.134	Mann-Whitney	0.103	0.260
	Subword TTR	0.694 ± 0.154	0.665 ± 0.148	Mann-Whitney	0.451	0.121

Note: Sex: proportion of female subjects. Other variables were represented as mean ± standard deviation. Effect sizes for group comparison on sex were indicated by contingency coefficient while others by Rank-Biserial Correlation. Statistic tests within this table were carried out with JASP 0.16.4.

### Semantic similarity analyses

Following previous studies [21,45], we employed two language models to estimate the semantic structures through semantic similarity scores. For the conceptual semantic space, we first tokenized the transcripts into words using spaCy (3.7.4, en_core_web_sm for English and el_core_new_sm for Greek). Stopwords (from the nltk package, majorly comprised of functional words), and punctuations were removed as they do not provide conceptual meaning, to focus on the meaningful and informative words. The embedding of each word was retrieved from fastText models pretrained in English and Greek respectively [48]. FastText provides static embeddings to words regardless of context. In contrast, contextual language models such as BERT [31], deliver contextual embeddings for the subwords, taking the preceding and succeeding contexts into account. Thus, while a word receives the same embedding from fastText regardless of its context, BERT encodes it within the broader context, yielding different embeddings if the context changes. BERT is thought to capture the hierarchical structure of language [32] and thus be able to represent grammar-sensitive meanings [21]. We used two monolingual BERT models in this study, one pretrained on English data (bert-base-uncased [31]) and another on Greek (nlpaueb/bert-base-greek-uncased-v1, [49]), to tokenize the transcripts and encode the tokenized texts into embeddings. Of note, BERT models tokenize a sentence into subwords, rather than words, to majorly avoid out-of-vocabulary problems. We did not remove any words for BERT analyses as they together formed an integral context. We first counted the quantity and type-token-ratio (TTR) of the tokenized transcript as general descriptive measures for the elicited speech, as reported in Table 1. TTR quantified the ratio of unique words (types) to the total number of words (tokens) in a text. For a tokenized and vectorized transcript $U:=\left(e_{1},e_{2},\ldots ,e_{n}\right)$, using either fastText or BERT, the local semantic similarity (LSim) is defined as averaged semantic similarity between consecutive words/subwords, as shown in equation (1):


$$\begin{array}{c} LSim:=\frac{1}{n-1}\underset{i=1}{\overset{n-1}{\sum }}\;cosine\_similarity\left(e_{i},e_{i+1}\right) \end{array}$$ (1)

where $e_{i}$ represents the vectorized embeddings of the tokens derived from the language models.

The global semantic similarity (GSim) is defined as averaged semantic similarity between every pair of two words/subwords, as shown in equation (2):


$$\begin{array}{c} \text{GSim:=}\frac{2}{n\left(n-1\right)}\underset{i=1}{\overset{n-1}{\sum }}\;\underset{j=i+1}{\overset{n}{\sum }}\;cosine\_similarity\left(e_{i},e_{j}\right) \end{array}$$ (2)

### Semantic perplexity analysis

Perplexity (PPL) indicates how perplexing a language model finds a text during sequential prediction. Given a sequence of tokens $\left(t_{1},t_{2},\ldots ,t_{n}\right)$, the differences between the predicted probability distribution and the true distribution (i.e., the actual tokens in the sequence) are computed as a cross-entropy loss, as shown in equation (3):


$$\begin{array}{c} H\left(p\right)=-\frac{1}{n}\underset{i=1}{\overset{n}{\sum }}\;log_{2}p\left(t_{i}\;\;\;|\;\;\;t_{1:i-1}\right) \end{array}$$ (3)

where $p\left(t_{i}\;\;\;|\;\;\;t_{1:i-1}\right)$ is the conditional probability of token $t_{i}$ given the preceding contexts $t_{1:i-1}$, and H(p) is the cross entropy of the probability distribution p. Perplexity is then defined as the 2-based exponentiation of the average negative log-likelihood H(p).

As the language models have been pretrained on data majorly from neurotypical populations, higher PPL indicates greater deviation and thus potentially higher abnormality in speech. PPL has been found to be a reliable speech coherence marker sensitive to cognitive decline, capturing meaning at the discourse level [40]. PPL is mainly defined for generative models, so masked models like BERT are not used for computing PPL. Rather, we estimated PPL using two state-of-the-art generative language models trained on the two target languages: a Mistral model with 7 billion parameters for English (mistralai/Mistral-7B-Instruct-v0.1 [50]) and one Greek model built on top of it (ilsp/Meltemi-7B-v1). The perplexity of each text was computed as the exponentiation of cross-entropy loss from the models.

### Bimodal semantic similarity

As the picture serves as an anchor in the generation of speech during the picture description task, tracing the similarity between elicited descriptions and the original pictures would inform how much the semantic space deviates from its anchor during the task. We used CLIP [43] to measure the similarity between the elicited descriptions and the original pictures. The images were encoded by CLIP with a large visual transformer (ViT-L/14) as its backbone. Transcripts were first segmented into sentences using the same spaCy models as stated above. English sentences were encoded by the original CLIP model while Greek sentences were encoded by a retrained multilingual model to expand CLIP to multilingual data (M-CLIP/XLM-Roberta-Large-Vit-L-14 [51]). For each subject, we first computed the similarity between every sentence and the original picture, and averaged the similarity scores to indicate the similarity between the whole description and the original picture.

### Formal syntactic complexity: Averaged dependency distance

To contextualize the differentiation in conceptual and referential semantic structure, we introduced one formal syntactic measure to explore the changes in formal syntactic hierarchy in AD. Every sentence in the speech was parsed with the dependency parser from the spaCy language models. Dependency parsing aims to find out the words with direct relationships and the type of such direct relationships in a sentence. For example, in *Mary ate the juicy red sweet apple*, *ate* is the *ROOT* of the sentence, while *Mary* is the *noun subject* of the *ROOT* verb, *apple* is the *noun object* of the *ROOT* verb with determiners and modifiers. Words with direct relationship (e.g., *ate* and *apple*) are called dependents. Distance between two dependents, as defined by the number of words in between, and its averaged mean across all word pairs, have been shown as an index for semantic control capacity [52], and contributed to the classification between AD and controls [53]. In this instance, the distance between *ate* and *apple* is 5. We extracted the dependency distance for every word pair in a sentence, excluding punctuations, and averaged them across all word pairs for averaged dependency distance (ADD) as the formal syntactic complexity indicator.

### Group difference comparisons and relationships to cognitive scores

Generalized linear models (GLMs) were employed to explore the group differences in semantic measures. As the semantic measures could be influenced by demographics, text length, and repetitions [21], we first assessed the correlations between semantic measures and potential covariates including age, sex, education, Type-Token Ratio (TTR), and word count, using Spearman partial correlation (covariates: language and group). Details on these correlations analysis were reported in S1 File. Results were adjusted using the False Discovery Rate (FDR) [54] to account for multiple comparisons and control the risk of false discoveries. The corrected *p* values were reported as *q* values. Correlation results are available in the supplementary material (Supplementary Figure 1 in S1 File). Variables exhibiting significant correlations (*q *< 0.05) were included in the GLMs. The universal formula for the GLMs in group comparison is:


$$\begin{array}{c} Measure\;\sim \;\beta_{0}+\;\beta_{1}*Group+\;\sum \;\beta_{i}*\;covariate_{i}\; \end{array}$$ (4)

GLMs offer a more flexible framework to accommodate response variables with error distribution models other than the normal distribution. For all measures, we first constructed generalized linear models with Gaussian distribution and Identity link function for all variables (equivalent to ordinary least squares regression), and tested whether such models fit the data well. Deviance goodness-of-fit tests indicated satisfactory fitting for all models except PPL, due to the positively skewed distribution. Thus, for PPL, the GLMs were fit with Gamma distribution and Log link function, which passed the deviance goodness-of-fit tests. All *p* values were corrected with FDR within each of the two datasets.

Furthermore, within the pAD groups, GLMs were constructed to examine the relationship between semantic measures and MMSE scores in AD pathology. All *p* values were corrected using FDR within each of the two datasets. The universal formula for the GLMs in relationship to MMSE scores is:


$$\begin{array}{c} Measure\;\sim \;\beta_{0}+\;\beta_{1}*MMSE+\;\sum \;\beta_{i}*\;covariate_{i}\; \end{array}$$ (5)

Statistical analyses were performed using Pingouin and Statsmodels with our custom-developed Python scripts. Statistical significance was recognized when the *p* value (or *q* value if corrected) was less than 0.05. Detailed results of the regression were reported in the S2 File.

### Crosslingual replication

All the regression models were first performed in the English data and then in the Greek data to test whether the result from English could be replicated in Greek or not. As the size of the Greek dataset is too small to expect similar effect sizes to emerge as in the English data, it would count as a satisfactory replication if the regression coefficients shared the same sign (i.e., no change in the direction of effect) and the regression coefficients from the English dataset fell in the confidence interval of the coefficients from the Greek datasets [55].

## Results

### Semantic space contracts in Alzheimer’s disease

**Fig 1:**
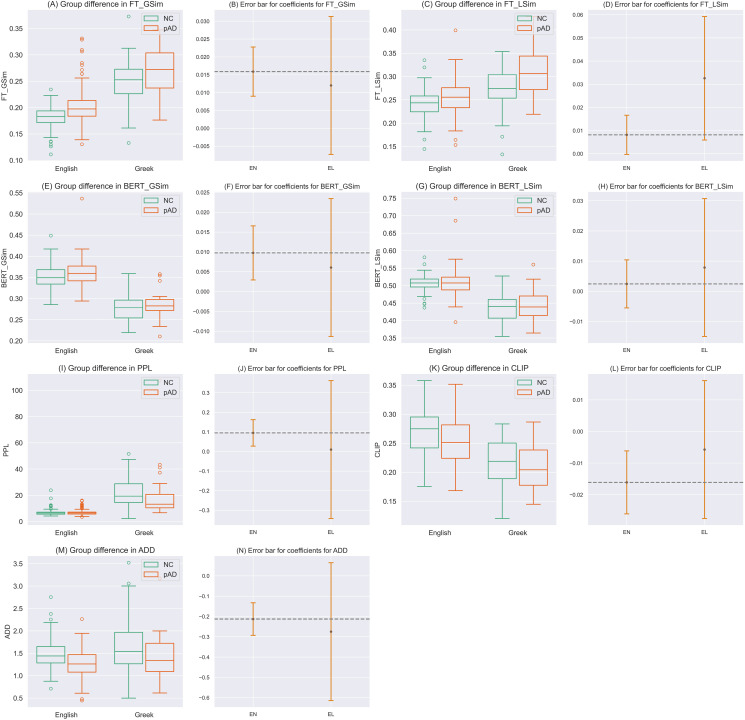
(A) Boxplots showing data distribution of global semantic similarity from fastText (FT_GSim) in HC (green) and pAD (orange) for English and Greek. **(B)** Error bar showing the regression coefficients (the central point) of the group in the generalized linear regression model predicting global semantic similarity from fastText and their 95% confidence intervals, for English and Greek data. The dash line indicates whether the regression coefficient from English data falls in the confidence interval of that computed from Greek data. If so, a replication is recognized. Same for the following error bar plots. **(C, D)** Boxplots and error bars of regression coefficients for local semantic similarity from fastText (FT_LSim). **(E, F)** Boxplots and error bars of regression coefficients for global semantic similarity from BERT models (BERT_GSim). **(G, H)** Boxplots and error bars of regression coefficients for local semantic similarity from BERT models (BERT_LSim). **(I, J)** Boxplots and error bars of regression coefficients for perplexity (PPL) from generative language models. **(K, L)** Boxplots and error bars of regression coefficients for CLIP-based similarity between the stimuli pictures and elicited speech. **(M, N)** Boxplots and error bars of regression coefficients for averaged dependency distance (ADD).

As shown in Fig 1A and 1B, compared to controls, conceptual global semantic similarity from fastText significantly increased in the pAD populations in English (*z* = 4.518, *p* = 0.000, *q *= 0.000), and insignificantly increased in Greek (*z* = 1.218, *p* = 0.223, *q *= 0.521). As shown in Fig 1C and 1D, conceptual local semantic similarity from fastText also increased, despite insignificantly, in pAD in both languages (English: *z* = 1.894, *p* = 0.058, *q *= 0.068; Greek: *z* = 2.396, *p* = 0.017, *q *= 0.116). Similarly, as depicted in the subplots E to H of Fig 1, increases in semantic similarity from BERT in pAD were found in English, significantly for the global similarity (*z* = 2.804, *p* = 0.005, *q *= 0.008) while insignificantly in local similarity (*z* = 0.606, *p* = 0.545, *q *= 0.545). Such increases in pAD, though insignificant, were found and replicated in Greek (global: *z* = 0.683, *p* = 0.494, *q *= 0.698; local: *z* = 0.676, *p* = 0.499, *q *= 0.698). These four measures, with satisfactory crosslingual replication, together suggest contracting semantic space at both levels of meaning, either sensitive to grammar or not, in pAD, with the contraction more prominent in the conceptual semantic space insensitive to grammar.

### Decrease of formal syntactic complexity

The dependency distance in pAD was significantly shorter in pAD in English (*z* = -5.189, *p* = 0.000, *q *= 0. 000), as compared to controls. Lower ADD was also replicated in the Greek data, though insignificantly (*z* = -1.584, *p* = 0.113, *q *= 0. 396).

### Perplexity of meaning in Alzheimer’s disease

The raw PPL score in pAD decreased in both English and Greek datasets, as shown in Fig 1I. However, such a decline was potentially a result of decrease in creativity as indexed by lower lexical diversity (i.e., lower TTR). When we regressed out the effects of TTR, group effects on PPL scores increased in pAD, significantly in English (*z* = 2.760, *p* = 0.006, *q *= 0.008) and insignificantly in Greek (*z* = 0.056, *p* = 0. 955, *q *= 0.955).

### Greater deviation from the visual prompts

As shown in Fig 1K and 1L, elicited speech was less aligned with the prompt pictures in pAD in the English dataset, as evidenced by the significant decrease in CLIP-based bimodal similarity (*z* = -6.163, *p* = 0.000, *q *= 0.000). This decrease was replicated, though insignificantly, in the Greek dataset (*z* = -0.622, *p* = 0.534, *q *= 0.623).

### Relationships to MMSE scores

**Fig 2:**
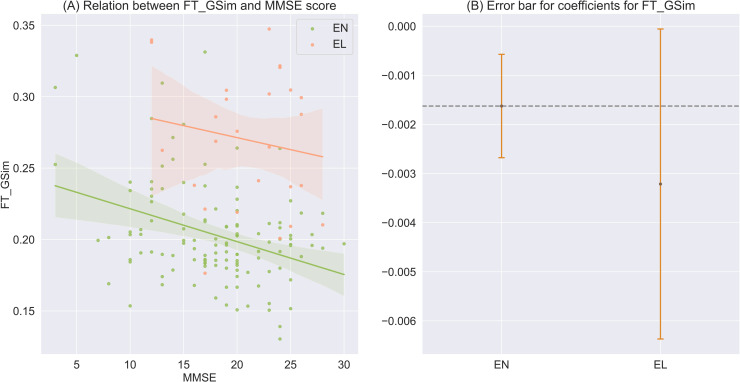
(A) Scatter plot with a fitting line of global semantic similarity from fastText and MMSE scores in English (green) and Greek (orange). **(B)** Error bar showing the regression coefficients (the central point) of MMSE score in the generalized linear regression model predicting global semantic similarity from fastText and their 95% confidence intervals, for English and Greek data. The dash line indicates whether the regression coefficient from English data falls in the confidence interval of that computed from Greek data.

As shown in Fig 2, in English, significant relationships were found between MMSE scores and global semantic similarity from fastText (negatively, *z* = -3.026, *p* = 0.002, *q *= 0.017). This relationship was successfully replicated in Greek data (*z* = -1.995, *p* = 0.046, *q *= 0.161). The contraction of conceptual semantic space indicated the general decline in the cognitive profiles in the AD spectrum.

## Discussion

In this paper, we aim to explore the semantic structure changes in AD pathology and validate the cross-lingual generalizability. The main findings can be summarized as follows: (a) Semantic space contracts in AD, both contextually and referentially; (b) this contraction coexists with significantly simplified formal syntactic hierarchy and the reduced distance from the ground truth (the picture to describe); and (c) only the contraction in conceptual semantic space is related to degeneration in the general cognitive profile. These patterns were observed mainly in the English dataset, but replicated in the Greek data to a certain level considering the overlapping of effect size.

Computing semantic similarity with word embeddings retrieved from language models has long been used to explore changes in spontaneous speech in multiple clinical populations, including schizophrenia [56–58], bipolar disorder [59], aphasia [60], and dementia [45]. However, these results have been typically interpreted as changes in semantic “coherence” [45,61–63]. However, recent research challenges this interpretation. For instance, studies in schizophrenia have shown increased averaged semantic similarity between adjacent words [21,57,58,64], despite individuals with schizophrenia typically producing less coherent speech [65–67]. A clear definition of coherence in the previous literature is lacking, such that the notion of coherence remains a primitive. In line with the proposal of He et al.[21], a more direct interpretation of these findings suggests that semantic similarity measures can unveil structural changes in semantic space triggered by visual stimuli. Our own investigation, focusing on individuals with probable Alzheimer’s disease (pAD), revealed a notable tightening of semantic space at both conceptual and referential levels of meaning. This tightening of the conceptual semantic space has been observed not only in the AD pathology, but also in psychotic populations [21,57,58,64], indicating that such contraction may reflect a decline in the general cognitive profile. Further supporting this notion, we found a relationship between the global semantic similarity and MMSE scores, providing additional evidence to the idea that the shrinking semantic space indexes general cognitive decline.

The contraction in conceptual semantic space has been demonstrated to have a neurological basis, reflecting the alternation in the distance between two ends of a large-scale cortical hierarchy from functional fMRI: the cortical visual network and the default mode network [20]. In other words, during picture description tasks, selecting concepts for discourse meaning construction can reflect how a participant makes sense of the visual stimuli. The visual language model provides a more direct approach to measuring such a distance between visual prompts and elicited speech, where we observed a decline in the pAD population. This dual pattern of decline in CLIP-based image-text similarity and increase in fastText-based word-to-word similarity could be an expected pattern in populations with cognitive decline. Notably, this dual pattern has also been observed in psychotic populations [21], and its neural basis (i.e., the altered functional distance between the visual and default mode networks) has been implicated in a wide range of conditions, including psychosis [68,69], autism [70], episodic migraine [71], and the AD spectrum [13,72,73]. Given its presence across these pathological states, this dual pattern may not be specific to a single condition like AD but could instead serve as a cross-pathological indicator.

Changes in the structure of the referential semantic space exhibited a distinct pattern compared to other pathologies, suggesting the referential semantic measures as potentially more specific disease markers than conceptual and bimodal semantic measures. In individuals with pAD, the referential semantic space also contracted, although not as significantly as the conceptual semantic space. This contraction coincided with changes in perplexity (PPL). In both datasets, the models found speech production from individuals with pAD to be as easy to understand as controls, and in some cases even slightly easier, albeit these changes were largely attributed to the lexical diversity of subwords (English: *p* = 1.15E-21, Greek: *p* = 1.82E-06), which decreased in pAD. The decline in creativity observed in populations with pAD limited their expressiveness, rendering their speech superficially as understandable as controls. However, when the effect of lexical richness was regressed out, the increase in PPL emerged notably. This suggests the strong impact of the organization of conceptual elements on the structure of referential meaning in pAD. Moreover, the contraction of referential semantic space in pAD cooccurred with the deterioration of formal syntactic complexity, indicating that the simplification of syntactic structure may also contribute to the contraction of the referential space. This can be further supported by the significant relations between ADD and the local semantic similarity from BERT and CLIP-based similarity, and no significant relations between ADD and measures derived from fastText, as reported in S1 File. In other words, the contraction of referential space in pAD could be a combinatory effect of two mechanisms: too closely situated conceptual elements and simplified structures to organize those elements.

In a nutshell, this study explored the contraction of semantic space in the pAD populations at both conceptual and referential levels. Shrinking conceptual semantic space co-occurred with less similarity between visual prompt and the elicited speech, which could be a cross-pathological marker for general cognitive decline. Contraction in the referential semantic space, as well as lower PPL due to decrease in lexical diversity, was less prominent than changes in the conceptual and bimodal measures, but potentially serve as a more specific marker to changes in the AD pathology. These findings were mainly observed in the English samples but also replicated to a certain degree in the Greek speech. As far as we know, this is the first study that compares and examines semantic changes in AD at both levels of conceptual and referential meanings using multiple different language models and derived measures with cross-lingual comparisons. However, it is noteworthy that our study had a relatively small sample size, as well as relatively short speech of around 30 or 50 words per speech, especially for the Greek dataset, which restricted the effect size of the regressions and limited the generalizability of the findings. This was compensated by the cross-lingual comparisons but still not satisfactorily solved. Future work could employ more and longer data from other languages to replicate the analyses. In addition, in these two datasets, we only have MMSE score as a cognitive state marker, without any clinical, neuropsychological, or biological marker specific to AD, like the visual processing capacity (relevant for the bimodal similarity), logical memory scores (for episodic memory loss) or pTau concentration (for biomarker changes). Subsequent analyses should explore whether some of these semantic measures, especially the referential measures, revealed changes specific to AD pathology or not.

## Supporting information

S1 File Appendix: Supplementary information on methods and figures. (DOCX)

S2 File Table: Regression results. (XLSX)

S3 File Linguistic features: Extracted features. (CSV)
