# Correction: The structure of spontaneous speech changes in Alzheimer’s disease: Crosslingual evidence from English and Greek

**DOI:** 10.1371/journal.pone.0351467

**Published:** 2026-06-09

**Authors:** Hong Jiang, Zhengwei Chen, Yu Liu, Chun Yang, Xiaofeng Yuan, Rui He

In the Funding statement, the grant number from the funder Department of Science and Technology of Guangdong Province is listed incorrectly. The correct grant number is: 2023A0505050118.
