# Supplementary material for: The structure of spontaneous speech changes in Alzheimer’s disease: Crosslingual evidence from English and Greek
Source: PLoS One. 2025 May 22;20(5):e0324270. doi: 10.1371/journal.pone.0324270 (PMC12097628; doi:10.1371/journal.pone.0324270)
Supplement: S1 File — (DOCX) [file pone.0324270.s001.docx]

Supplementary Material

## Regression models

As shown in Supplementary Figure 1, all demographic factors, except for sex in relation to global similarity from fastText, were not included in the regression models. Local and global semantic similarity from fastText incorporated the Type-Token Ratio (TTR) of words (excluding stopwords) as a covariate. ADD incorporated the number of words (excluding stopwords) as a covariate. Global semantic similarity from BERT and CLIP-based text-image similarity utilized the number of subwords as a covariate, while local similarity from BERT did not employ a covariate. PPL from generative language models incorporated the Type-Token Ratio (TTR) of subwords as a covariate. For measures correlating to more than one word count or TTR measures, we only included one of them as a covariate, as these four measures were strongly correlated with each other. Including more than one measure could result in multicollinearity.

## Supplementary Figures


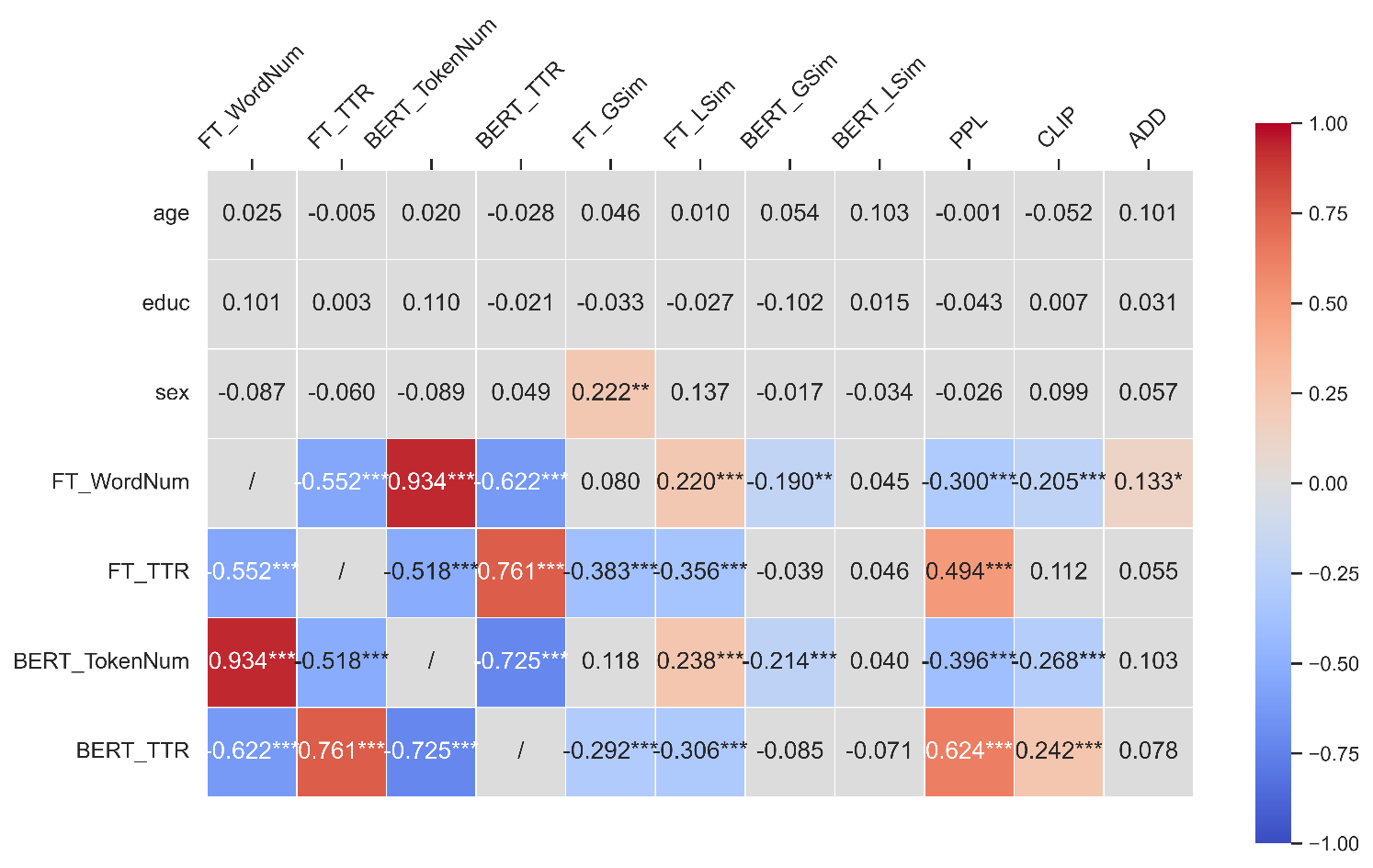


**Supplementary Figure 1.** Correlation between semantic measures (on the top) and descriptive variables (on the left). Each cell represents the correlation coefficients between them. Only cells with statistical significance were colored. Blue cells indicate negative correlations while orange cells indicate positive correlations. The number in the cell is the correlation coefficient. * q < 0.05, ** q < 0.01, *** q < 0.001**.**


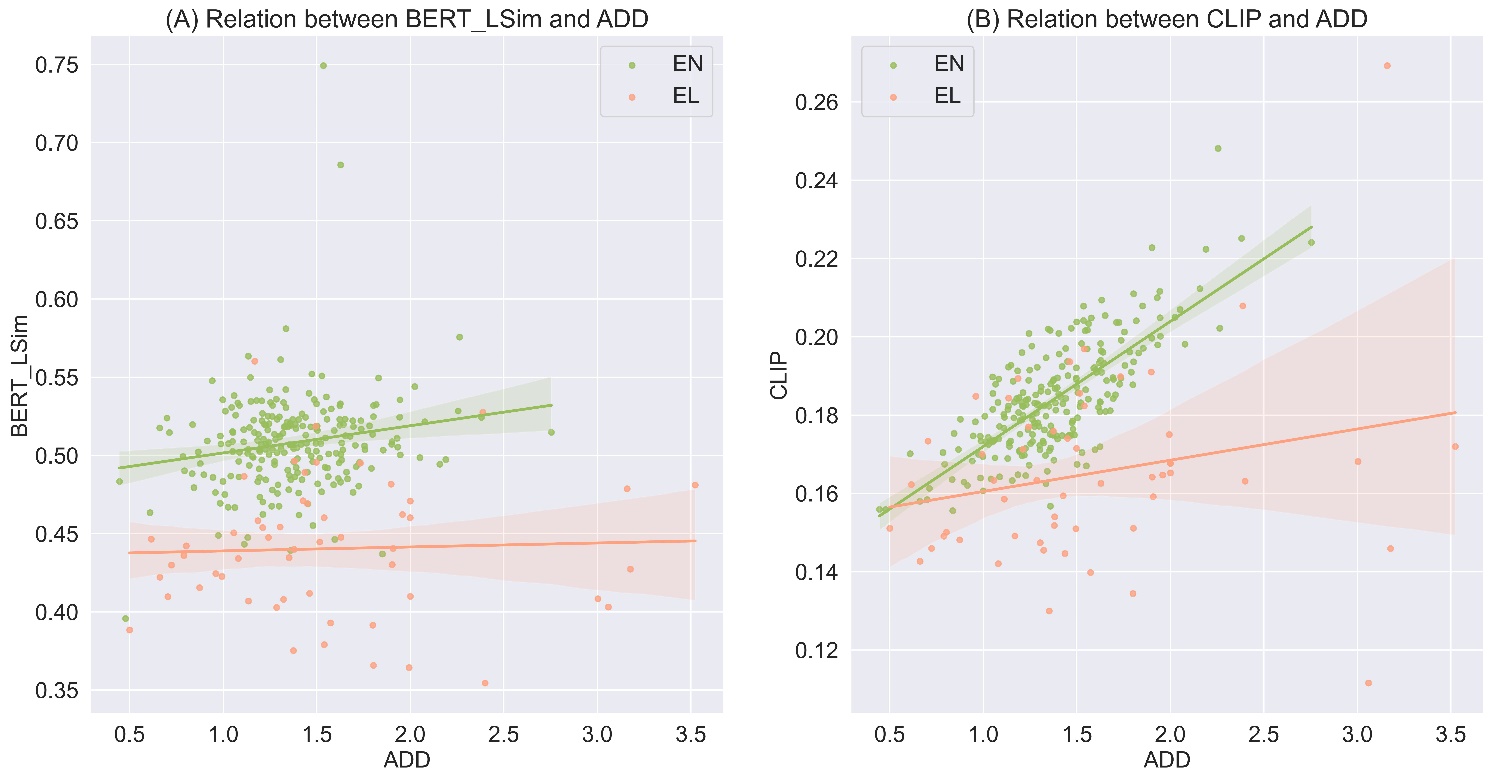


**Supplementary Figure 2.** (A) Scatter plot with a fitting line of local semantic similarity from BERT and ADD in English (green) and Greek (orange), with diagnosis group as a covariate. (B) Scatter plot with a fitting line of CLIP-based similarity and ADD in English (green) and Greek (orange), with diagnosis group as a covariate.
